# Differential expression of mechanotransduction complex genes in auditory/vestibular hair cells in zebrafish

**DOI:** 10.3389/fnmol.2023.1274822

**Published:** 2023-11-14

**Authors:** Eliot T. Smith, Peng Sun, Shengyang Kevin Yu, David W. Raible, Teresa Nicolson

**Affiliations:** ^1^Department of Otolaryngology-HNS, Stanford University, Stanford, CA, United States; ^2^Department of Otolaryngology-HNS and Biological Structure, Viginia Merrill Bloedel Hearing Research Center, University of Washington, Seattle, WA, United States

**Keywords:** *tmc1*, *tmc2a*, *tmc2b*, *cib2*, *cib3*, hair cell, inner ear, mechanotransduction

## Abstract

Ciliated sensory cells such as photo- and olfactory receptors employ multiple types of opsins or hundreds of unique olfactory G-protein coupled receptors to respond to various wavelengths of light or odorants. With respect to hearing and balance, the mechanotransduction machinery involves fewer variants; however, emerging evidence suggests that specialization occurs at the molecular level. To address how the mechanotransduction complex varies in the inner ear, we characterized the expression of paralogous genes that encode components required for mechanotransduction in zebrafish hair cells using RNA-FISH and bioinformatic analysis. Our data indicate striking zonal differences in the expression of two components of the mechanotransduction complex which are known to physically interact, the *transmembrane channel-like 1* and *2* (*tmc1/2*) family members and the *calcium and integrin binding 2* and *3* (*cib2/3*) paralogues. *tmc1*, *tmc2b*, and *cib3* are largely expressed in peripheral or extrastriolar hair cells, whereas *tmc2a* and *cib2* are enriched in central or striolar hair cells. In addition, a gene implicated in deaf-blindness, *ush1c*, is highly enriched in a subset of extrastriolar hair cells. These results indicate that specific combinations of these components may optimize responses to mechanical stimuli in subtypes of sensory receptors within the inner ear.

## Introduction

The inner ear senses a wide range of mechanosensory stimuli in terms of frequency and intensity. Sensitivity of the sensory receptors known as hair cells relies in part on various attributes and specializations. Factors that influence sensitivity include local external structures, position of the hair cells within cochlea or location in central “striolar” zones versus peripheral “extrastriolar” zones in vestibular organs, and the architecture of the apical hair bundle of cilia (Ó Maoiléidigh and Ricci, 2019; Nam et al., 2019). Recent evidence suggests that the mechanotransduction apparatus, which consists of a “tip link” extracellular filament that is anchored at the upper site and interacts with a channel complex at the low end, is also specialized. Genetic and molecular studies in several species indicate that the mechanotransduction channel complex consists of several transmembrane proteins, including the TMC1/2 subunits, TMIE, CIB2/3, LHFPL5, and PCDH15, whereas the upper transduction complex includes CDH23 and USH1C (reviewed in Nicolson, 2017; Holt et al., 2021; Caprara and Peng, 2022; Qiu and Müller, 2022). In some cases, these components may vary in terms of the paralogues (TMC1 vs. TMC2 or CIB2 vs. CIB3) or splice variants (e.g., PCDH15) expressed (Kawashima et al., 2011; Webb et al., 2011; Giese et al., 2017). For example, TMC1 is expressed in mature cochlear hair cells in mice, whereas TMC2 is not necessary for hearing (Kawashima et al., 2011). In zebrafish there is one *tmc1* gene and two paralogues (also called ohnologues) of *tmc2*, *tmc2a*, and *tmc2b*, which were duplicated in teleost fish during evolution (Maeda et al., 2014). Functional differences have been described for the zebrafish inner ear and lateral line in terms of reliance on the *tmc1/2* genes (Chou et al., 2017; Chen et al., 2020; Smith et al., 2020; Zhu et al., 2021; Kindig et al., 2023). In the inner ear, the function of both the zebrafish larval utricle and saccule relies largely on *tmc2a* and *tmc2b* (Chen et al., 2020; Smith et al., 2020; Zhu et al., 2021). In addition, differences in labeling with vital dyes used as a proxy for mechanotransduction were observed within the cristae of various *tmc1/2* mutants, with one subtype showing a preference for *tmc2a* (Smith et al., 2020; Zhu et al., 2021). These functional differences are suggestive of differential expression of these genes in zebrafish, however, the spatiotemporal pattern of expression in each sensory end organ has not been reported for the *tmc1/2* paralogues. In addition, whether switching among TMC paralogues during development is a conserved feature in the vestibular labyrinth or in vertebrates in general is not clear. Moreover, whether various components of the mechanotransduction complex preferentially coalesce into specific combinations within distinct *TMC1/2* subtypes of hair cells has not been fully explored.

Here we characterize the expression pattern of the *tmc1/2* orthologues in the zebrafish inner ear during development. The fish inner ear resembles the labyrinth in other species, with five sensory or “end” organs: the macular end organs of the utricle and saccule along with three cristae in the semicircular canals (Baeza-Loya and Raible, 2023). In contrast to mammals, hair cells within the maculae mature quite rapidly in zebrafish. Extracellular potentials (microphonics) in response to mechanical stimuli are detectable as early as 2 days postfertilization (dpf; Lu and DeSmidt, 2013). The posterior macular organ, which is destined to become the saccule, is the primary source of microphonic signals at this stage of development (Inoue et al., 2013) and both fish and frogs use the saccule for hearing. At 27 h postfertilization (hpf), hair cell-mediated responses in third order neurons in the hindbrain are already present (Tanimoto et al., 2009). In addition, reflexive movements of the eye can be induced by low frequency head movements at 3 dpf (Mo et al., 2010; Bianco et al., 2012). Due to the rapid development of hair cell function, we included embryonic stages when hair cells first appear in the developing macular end organs of the inner ear. We also characterized later developmental stages of 5 and 15 dpf to determine whether the pattern changed over the course of time. Our data indicates that all three *tmc* genes are expressed at early stages with subsequent *tmc* subtypes of hair cells emerging between 2 and 5 dpf. After 5 dpf, the patterns no longer vary. Our previous study using scRNAseq data from whole zebrafish indicated that distinct hair cell types emerge in macular organs during larval stages (Shi et al., 2022). We leveraged this data set to further explore the expression of other mechanotransduction components within *tmc* subtypes. We found specific expression patterns of *cib2* and *cib3*, which encode calcium binding proteins that interact with TMC1/2 subunits and modulate channel conductance (Liang et al., 2021; Giese et al., 2023; Wang et al., 2023). Our data indicate that specialization of the mechanotransduction apparatus occurs at the molecular level.

## Materials and methods

### Ethics statement and fish line

Zebrafish (*Danio rerio*) were maintained and bred using standard procedures and animal research complied with guidelines of Laboratory Animal Care and Use stipulated by Stanford University. The wild-type zebrafish line used for this study was Topfin Long. All embryos and larvae were 1–15 dpf, before sex determination is possible.

### RNA-FISH

The Hybridization Chain Reaction (HCR) assay was used to detect various mRNAs in whole-mount samples (HCRv3; Molecular Instruments). DNA oligonucleotide probes targeting the genes in this study were designed by Molecular Instruments and used per the manufacturer’s instructions. Each probe was assigned to a separate fluorophore channel to enable multiplex detection in each specimen.

Embryos and larvae were raised in 1× E3 with 0.003% PTU starting at 20–24 hpf to prevent pigmentation, and E3 + PTU was replaced daily. Embryos and larvae were collected, anesthetized, and fixed overnight in 4% *p*-formaldehyde (PFA) in 1× PBS at 4°C (n ≥ 8 for each stage). After fixation, specimens were rinsed in PBS, dehydrated and permeabilized with 100% methanol, and stored in 100% methanol at −20°C until use.

RNA-FISH followed the official protocol for HCR in whole-mount zebrafish, from the manufacturer. Briefly, specimens were rehydrated from methanol into PBST by stepwise, 5-min incubations in PBST with 75, 50, and 25% methanol, followed by five incubations in PBST. Rehydrated samples were permeabilized with 30 μg/ml proteinase K in PBST at room temperature for 15–20 min. Specimens were then rinsed with PBST, post-fixed in 4% PFA in 1× PBS for 20 min, and rinsed 5× for 5 min in PBST.

Specimens incubated in pre-hybridization buffer for 30 min at 37°C prior to addition of the probes. A pooled solution of the probes was prepared in probe hybridization buffer as described in the manufacturer’s protocol. Specimens were incubated in probe hybridization buffer with probes overnight at 37°C. The next day, specimens were washed 4× for 15 min in probe wash buffer and then 2× for 5 min in 5× SSCT at room temperature.

Amplification hairpin oligonucleotides were prepared and applied as described in the manufacturer’s protocol (HCRv3; Molecular Instruments). Individual hairpin h1/h2 solutions were denatured at 95°C for 90 s and cooled to room temperature prior to pooling in amplification buffer. Specimens incubated overnight in the dark at room temperature in the hairpin solution. Hairpin solution was removed by multiple wash steps in 5× SSCT and stored at 4°C in the dark prior to imaging.

### Imaging

All imaging was performed with LSM700 laser-scanning confocal microscopes (Carl Zeiss). Three-dimensional views of neuroepithelia were generated using Imaris software; two-dimensional views were generated using ZEN (Carl Zeiss) and Image J software. For all stages, more than 8 embryos or larvae were examined in at least two independent experiments.

### Single-cell RNAseq data processing

The single-cell RNA sequencing dataset that was utilized for this portion of the analysis comes from the Danio-cell database (Sur et al., 2023). The data can be download via GEO with accession number: GSE223922. This dataset provided the raw data count matrix which was then processed in R via the Seurat Package (v4; Stuart et al., 2019). Based on existing clustering labels, the inner ear portion of the atlas was isolated and then converted to an h5ad format for further analysis in Python via the scanpy package (Wolf et al., 2018). Standard downstream pre-processing steps were taken to log normalize the data (sc.pp.normalize_total, sc.pp.log1p). UMAPs were generated through creation of PCA dimensional reduction to 40 components. Additional analysis tools such as differential gene expression were employed via “*t*-test” through scanpy’s function: rank_genes_groups while the rest of the data manipulation was done via other scanpy package functions. See full notebook code here: https://colab.research.google.com/drive/1lP2npK34-T5qWeb1yztSLncf4tZ9Pyr5?usp=sharing.

## Results

### Emergence of distinct *tmc1/2* subtypes during early development

**Figure 1:**
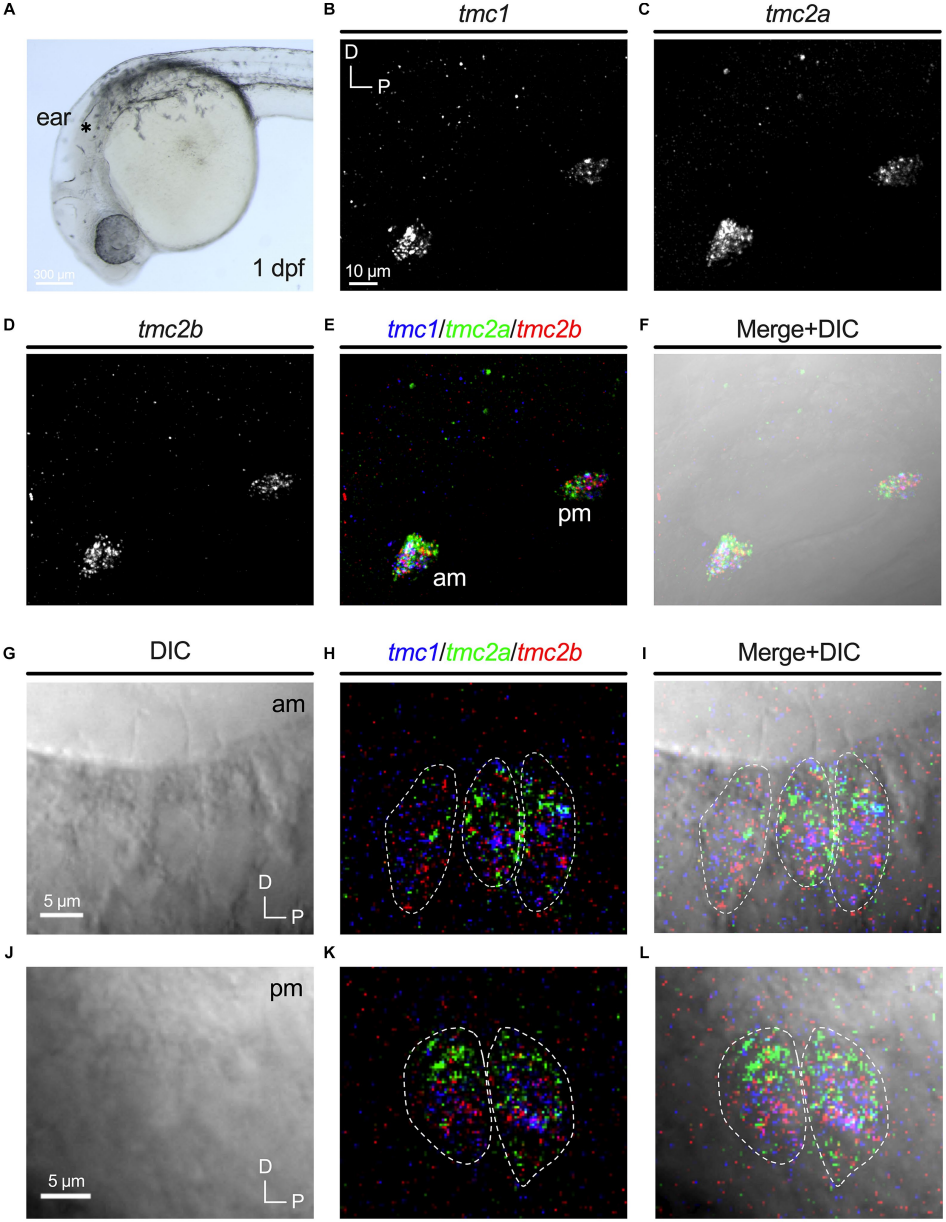
Overlapping expression of *tmc1/2a/2b* in nascent hair cells at 1 dpf. **(A)** Image of the anterior region of a 1 dpf embryo. Otocyst indicated by asterisk. Expression of *tmc1* **(B)**, *tmc2a* **(C)**, and *tmc2b* **(D)**. Merge of all three channels **(E)** and overlay with a DIC image **(F)**. In merged images, fluorescent label for *tmc1* transcripts is shown in blue, *tmc2a* in green, and *tmc2b* in red. **(G–L)** Single optical sections (0.75 μm) of overlapping expression of *tmc1/2a/2b* in hair cells at 1 dpf. Dotted outlines indicate the somas of single hair cells. **(G–I)** Lateral view of three hair cells in the anterior macula at 1 dpf with visible apical structures. **(J–L)** Lateral view of the cell bodies of two hair cells in the posterior macula at 1 dpf (kinocilia are not present within the same section).

**Figure 2:**
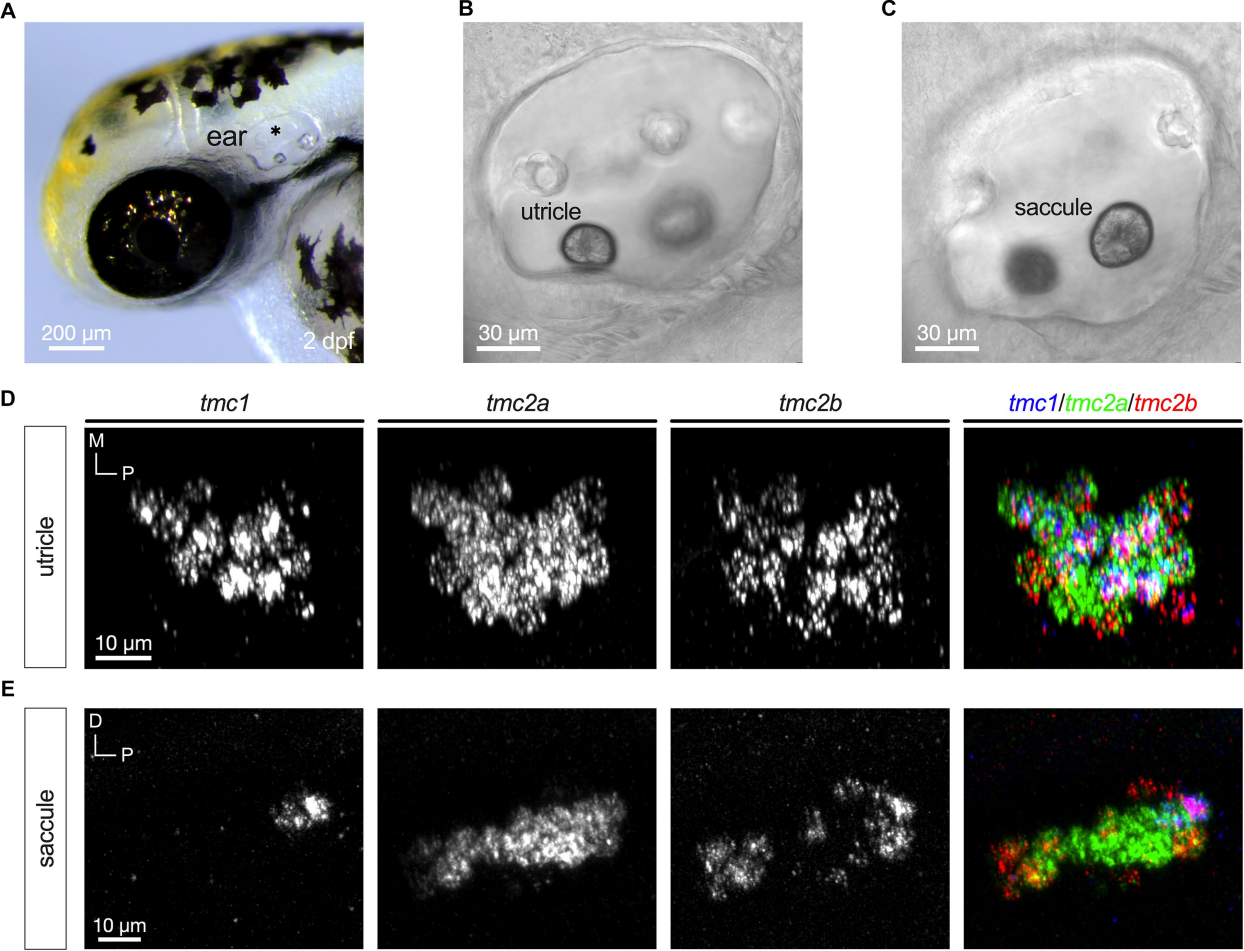
Emergence of differential expression of *tmc1/2a/2b* at 2 dpf. **(A)** Image of the anterior region of a 2 dpf embryo (asterisk indicates otocyst). Lateral views with focal planes including the anterior macula (utricle; **B**) and posterior macula (saccule; **C**) are shown. **(D)** Maximum projection each *tmc* gene including a merge of all three genes expressed in a top-down view of the anterior macula (utricle; right panel). **(E)** Expression of *tmc1/2a/2b* in the posterior macula (saccule). In merged images, fluorescent label for *tmc1* transcripts is shown in blue, *tmc2a* in green, and *tmc2b* in red.

Nascent hair cells in the developing otic capsule first appear approximately at 18 hpf (Whitfield, 2020). By 24 hpf, four to six hair cells are present in two macular organs, which include a tiny otolith (Figure 1A). The anterior macula will develop into the utricle and the posterior macula will become the saccule. We examined expression of *tmc1*, *tmc2a*, and *tmc2b* transcripts in 1 dpf specimens using HCRv3 RNA-FISH (Figures 1B–D). At this early stage, expression of all three genes overlaps in all hair cells (Figures 1E,F). To further corroborate the initial phase of expression of all three *tmc* transcripts, we examined single optical sections of hair cells in both maculae at 1 dpf (Figures 1G–L) In single optical sections, triple labeling was detected in all hair cells (*n* = 17 hair cells). One day later at 48 hpf when the number of hair cells has more than doubled in each macula (Figures 2A–C), a mixed pattern exists with most hair cells still expressing two or three of the *tmc* paralogues along with a small subset of hair cells expressing just a single gene, *tmc2a* (Figures 2D,E). The emergence of hair cells expressing *tmc2a* alone is particularly noticeable in the central region of the saccule (Figure 2E). These results suggest that early-stage hair cells express all three *tmc1/2* genes and gradually transition to assorted patterns of expression.

**Figure 3:**
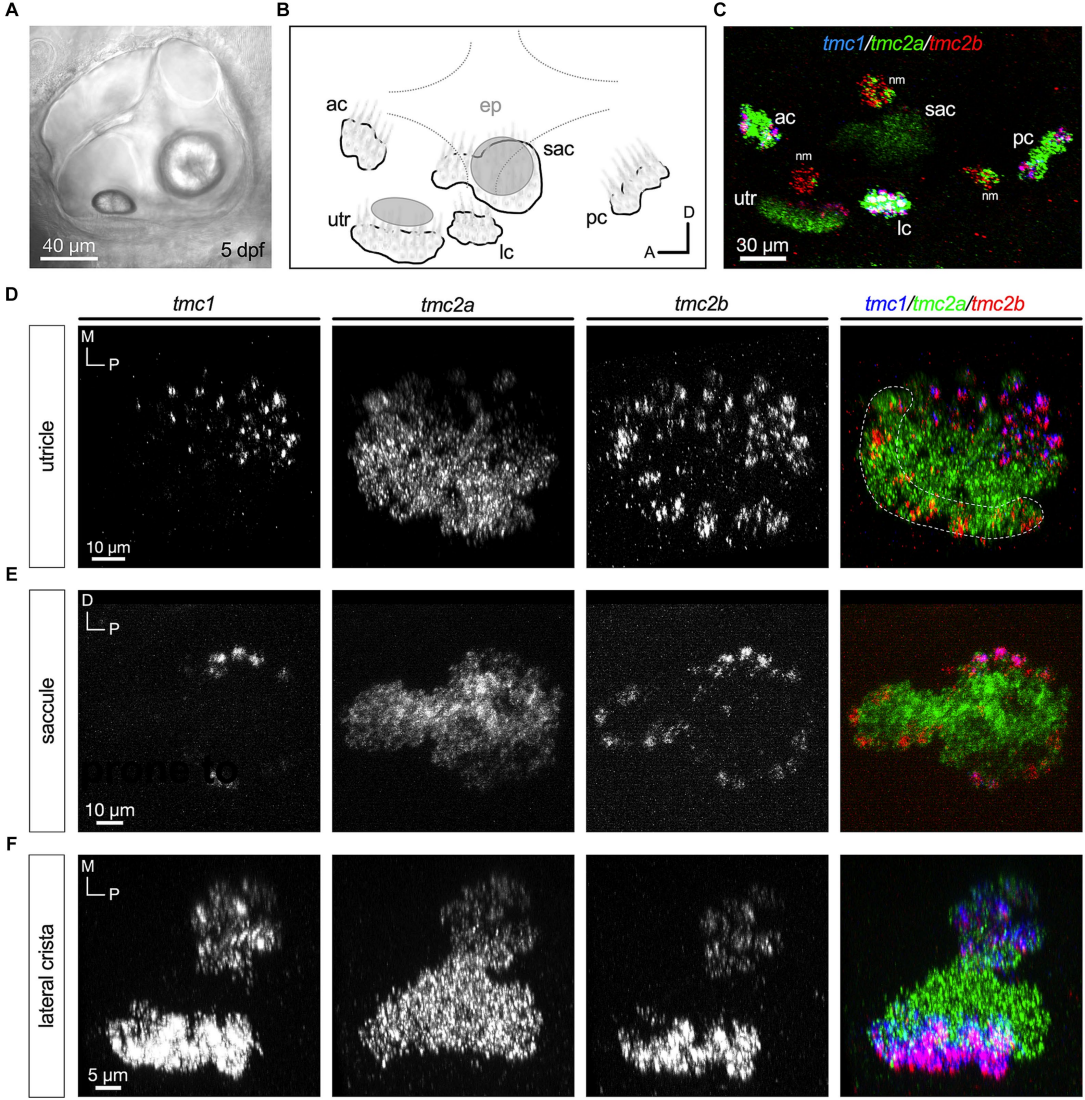
Central zones of *tmc2a* with *tmc1/2b* in peripheral hair cells at 5 dpf. **(A)** Lateral view of the inner ear. **(B)** Schematic of the five end organs at this stage of development. **(C)** Overview of *tmc1/2a/2b* expression in the ear including three overlying neuromasts at the surface of the skin. **(D–F)** All are top-down views of each end organ. Single-channel fluorescence for each gene is depicted, along with merged images. In merged images, fluorescent label for *tmc1* transcripts is shown in blue, *tmc2a* in green, and *tmc2b* in red. The presumptive striolar zone of the utricle is outlined in **(D)**.

We then examined expression at 5 dpf, which is a free-swimming larval stage of development where postural control and acoustic startle reflexes are robust (Figure 3). At this stage, cristae in the developing semicircular canals are present, although behavioral responses mediated by this cell type are not apparent until 25 dpf (Beck et al., 2004). Figures 3A,B shows a lateral view of the inner ear with a schematic of the five sensory end organs. The inferred striolar region of the utricle at 5 dpf was added based on previous publications (Hammond and Whitfield, 2006; Inoue et al., 2013; Liu et al., 2022). At 5dpf, the predominance of hair cells expressing *tmc2a* alone is more evident (Figures 3C–F). In each end organ, *tmc2a* is expressed throughout the neuroepithelium (Figures 3D–F). In contrast, *tmc1* expression is restricted to the medial extrastriolar region of the larval utricle and peripheral cells in the larval saccule and cristae (Figures 3D–F). This pattern is consistent with previous findings showing that vital dye labeling of hair cell somas remains in the posterior lobe of the posterior macula in *tmc2a/2b* mutants (Smith et al., 2020; Zhu et al., 2021). The *tmc2b* paralogue varies according to end organ. In the larval utricle, *tmc2b* is expressed in *tmc1*+ cells within the extrastriola and in addition, *tmc2b* transcripts are observed in a subset of *tmc2a*+ cells in the striola (Figure 3D). In the larval saccule and crista, *tmc2b* is predominantly expressed in peripheral hair cells, either outlining the entire saccule or at both ends of the cristae (Figures 3E,F). In addition, *tmc2b* was seen in a few hair cells with more central locations within the macular organs.

**Figure 4:**
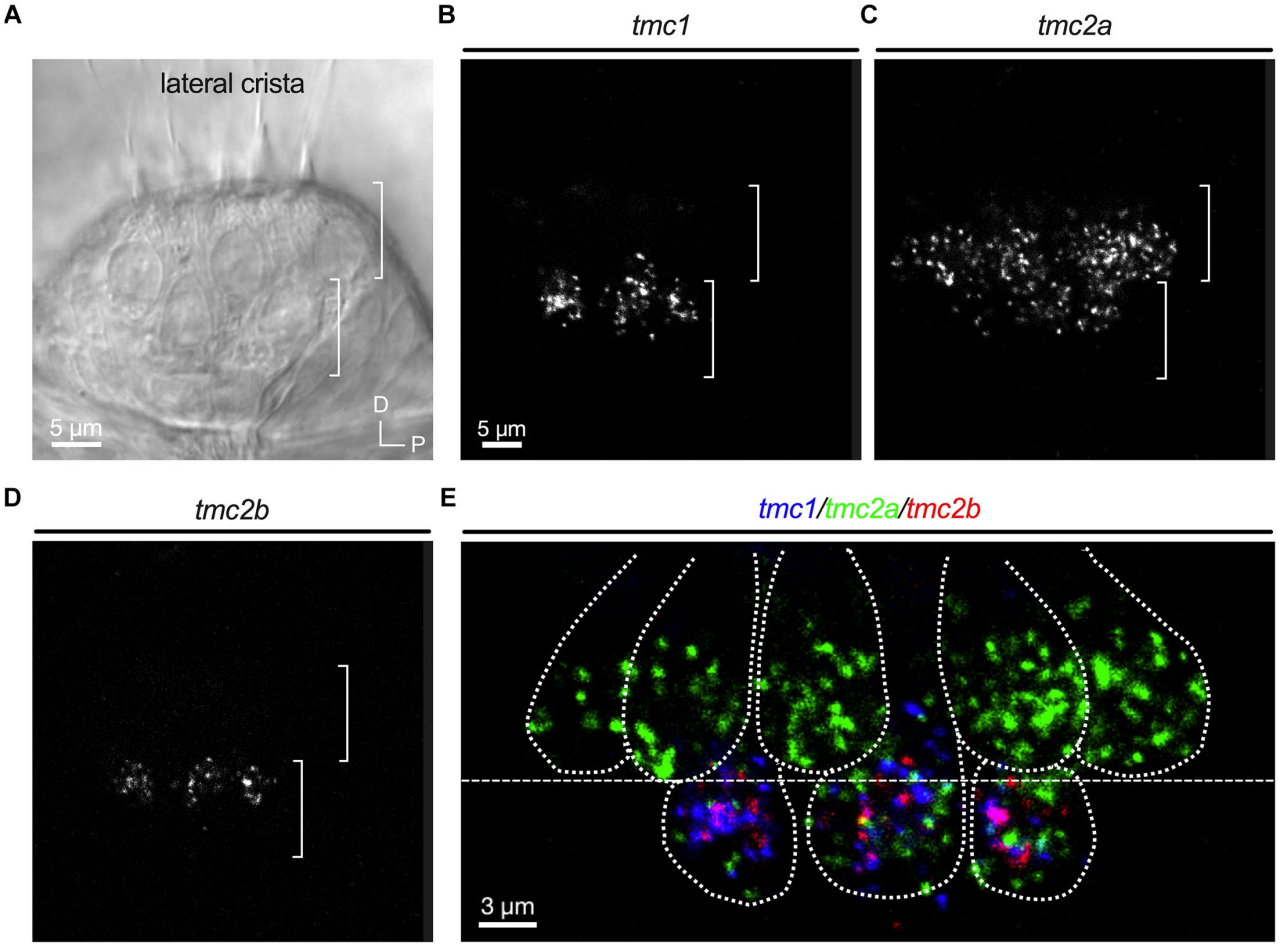
Differential expression in hair cell layers of the lateral crista at 5 dpf. **(A)** Lateral view of the lateral portion of the crista. Layers are indicated by brackets. **(B–D)** Single-channel fluorescence for each *tmc* gene. **(E)** Merge indicating expression of *tmc2a* in the upper layer and expression of all three *tmc1/2* genes in the lower layer of hair cells. Dotted lines outline cell bodies. In merged image, fluorescent label for *tmc1* transcripts is shown in blue, *tmc2a* in green, and *tmc2b* in red.

Although the neuroepithelia of larval macular end organs appear to have single layers of hair cells, regions of the cristae exhibit two layers of hair cells that are reminiscent of the pattern in other species (Lysakowski and Goldberg, 2008). As previously reported (Smith et al., 2020; Zhu et al., 2021), round, tear drop shaped hair cells are present in an upper layer and gourd shaped hair cells with nuclei positioned in a lower layer (Figure 4A). The upper layer hair cells show a dependence on *tmc2a* function in terms of vital dye labeling, whereas the lower layer is *tmc1/2b* dependent (Smith et al., 2020; Zhu et al., 2021). To determine whether differential expression extends to these two layers, we examined optical cross sections of the lateral crista (Figures 4B–D; *n* = 4 cristae). Our data indicate that the top layer largely expresses *tmc2a*, whereas the bottom layer of hair cells expresses all three *tmc* paralogues (Figure 4E).

### Differential expression of *tmc1/2* paralogues persists during development

**Figure 5:**
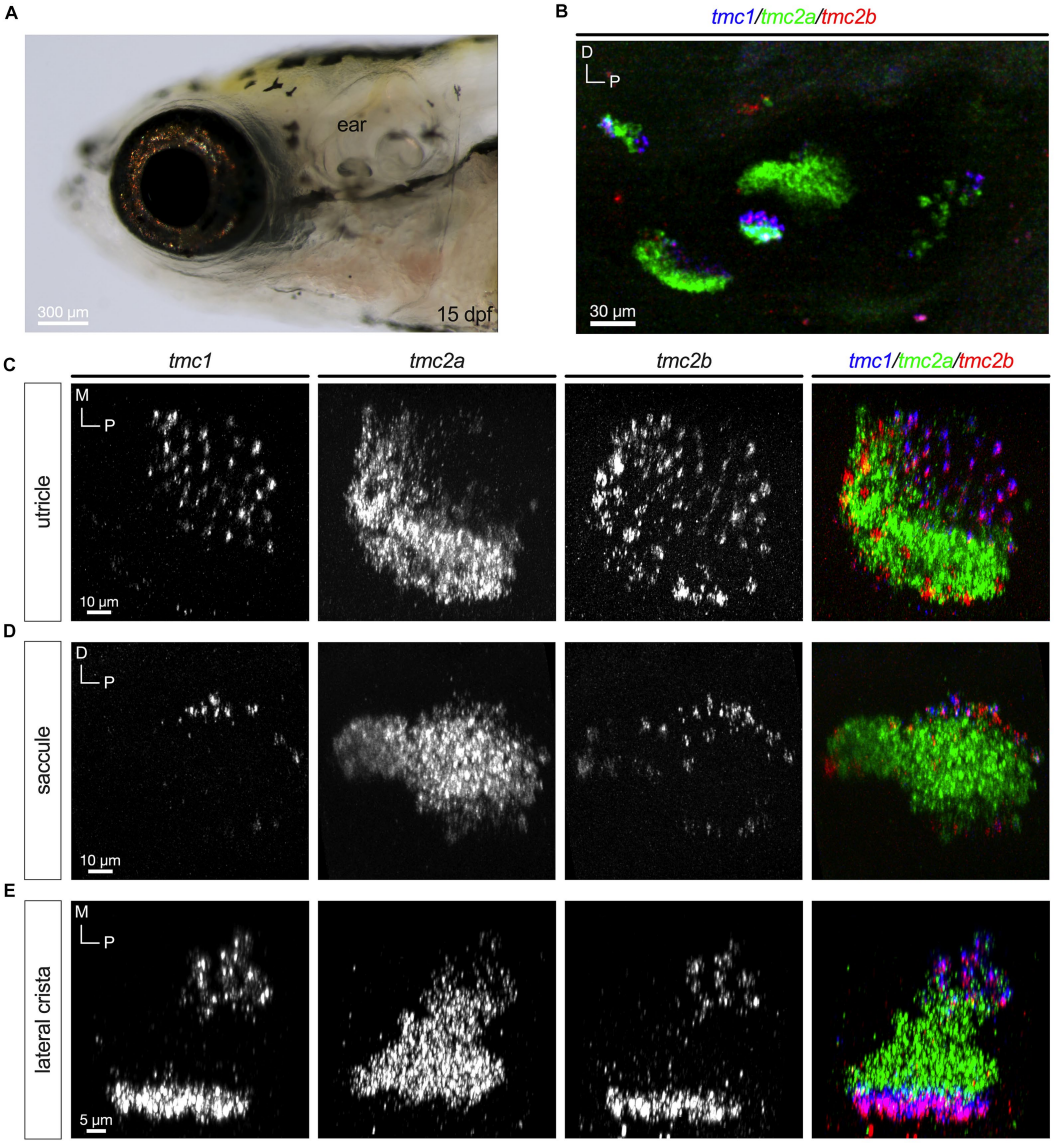
Differential pattern of expression of *tmc1/2a/2b* persists at 15 dpf. **(A)** Lateral view of the inner ear. **(B)** Overview of *tmc1/2a/2b* expression in the ear. **(C–E)** Single-channel fluorescence for each gene is shown along with merged images (right panels). In merged images, fluorescent label for *tmc1* transcripts is shown in blue, *tmc2a* in green, and *tmc2b* in red.

In the mouse cochlea, hair cells transition from expressing *Tmc2* to *Tmc1* at more mature stages (Kawashima et al., 2011). To determine whether the patterns observed at early larval stages were transient or if they persisted to later stages of development, we examined expression of *tmc1/2* transcripts at 15 dpf (Figure 5A). We labeled whole mounts using the same HCRv3 method as was done for earlier stages (Figure 5B). We found that the general pattern of *tmc2a* transcripts being expressed predominantly in the lateral region of the utricle and central regions of each end organ was nearly identical to the patterns seen at 5 dpf (Figures 5C–E). In addition, the restriction of *tmc1* to peripheral hair cells was similar. The pattern of *tmc2b* was also largely unchanged. One notable exception was the addition of what appeared to be another row of *tmc2b*+ positive cells formed near the lateral edge of the utricle (Figure 5C).

### Differential expression of other mechanotransduction components

**Figure 6:**
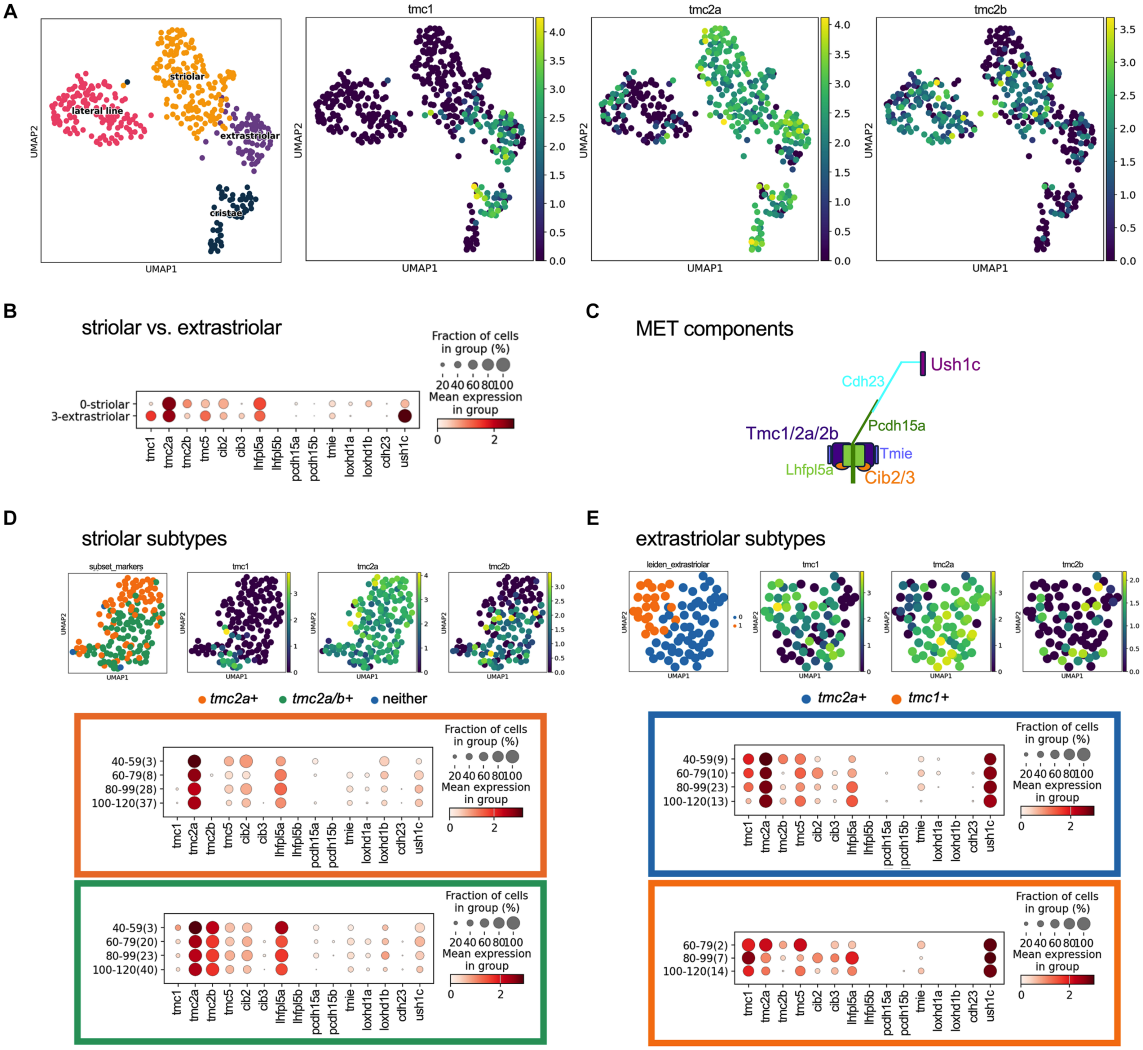
Expression of *tmc1/2a/2b* and mechanotransduction components in hair cells at 40–120 hpf using scRNAseq data. **(A)** UMAP distributing the striolar, extrastriolar, lateral line and ampullary/cristae inner ear hair cells with expression of *tmc1/2a/*2b with log-normalized scRNAseq expression output. **(B)** Dotplot comparing presence and expression of these components in striolar vs. extrastriolar hair cells of the macular organs. **(C)** Diagram of the components that comprise the mechanotransduction complex. **(D)** UMAP of the striolar subset and clustered to *tmc2a* + versus *tmc2a/2b* + with dotplot showing the expression distribution of MET channel components for the *tmc2a* + (orange) compared to *tmc2a/2b* + (green). **(E)** UMAP of the extrastriolar subset and clustered to *tmc2a* + versus *tmc1*+ with dotplot showing the expression distribution of mechanotransduction channel components for the *tmc2a* + (blue) compared to *tmc1*+ (orange). In the Y-axis of the dotplot, the age of the cell is grouped in hours with the number of samples in the group labeled in parenthesis.

To determine whether other genes encoding components of the mechanotransduction apparatus were universally or differentially expressed like the *tmc1/2* genes, we analyzed the transcriptional profile of key genes in specific end organs. Utilizing the daniocell single cell RNA sequencing dataset (Sur et al., 2023), we mapped the distribution of *tmc* genes within the inner-ear hair cells. We identified 528 hair cells from 40 hpf to 120 hpf and further classified cells based on known transcriptomic markers. We identified 136 hair cells as lateral line hair cells while for the inner ear, we identified 165 striolar hair cells and 78 extrastriolar hair cells (Figure 6A). We used previously identified markers for these distributions: *cabp2b* and *pvalb9* for striolar hair cells and *zgc:153395* (*skor2*) and *cabp1b* for extrastriolar hair cells (Shi et al., 2022). Based on these classifications, the single cell data correlate well with the mRNA *in situ* analysis, showing that *tmc* transcripts describe unique subpopulation of cells in the stages of development. We observed that *tmc1* expression is predominantly in the extrastriolar hair cells, while there is expression of *tmc2a* in both striolar and extrastriolar populations.

With the single cell data, we further explored the expression of other known mechanotransduction components in these cells (Figure 6C). We compared the expression of these corresponding transcripts in the striolar and extrastriolar populations (Figures 6B,C). The subpopulations of the striolar hair cells could be further separated into *tmc2a*+ and *tmc2a/2b*+ subtypes (Figure 6D) and the extrastriolar hair cells could be grouped into a predominantly *tmc2a*+ population versus a *tmc1*+ subpopulation (Figure 6E).

To confirm the above findings, we examined the expression of candidate genes that were differentially expressed among the various subtypes of hair cells using RNA-FISH. We used 5 dpf larvae for our experiments as the *tmc1/2* pattern did not vary considerably after this stage. Candidates with the most striking differences in the expression dot plots in Figures 6D,E include *cib2* and *cib3*, which encode calcium binding proteins that are known to interact with the TMCs in other species (Giese et al., 2023; Wang et al., 2023), *loxhd1a* and *loxhd1b*, which encode multi-PLAT domain proteins whose orthologue in mice is implicated in mechanotransduction in cochlear hair cells (Trouillet et al., 2021), and *ush1c*, encoding a component of the upper tip link (Grillet et al., 2009).

**Figure 7:**
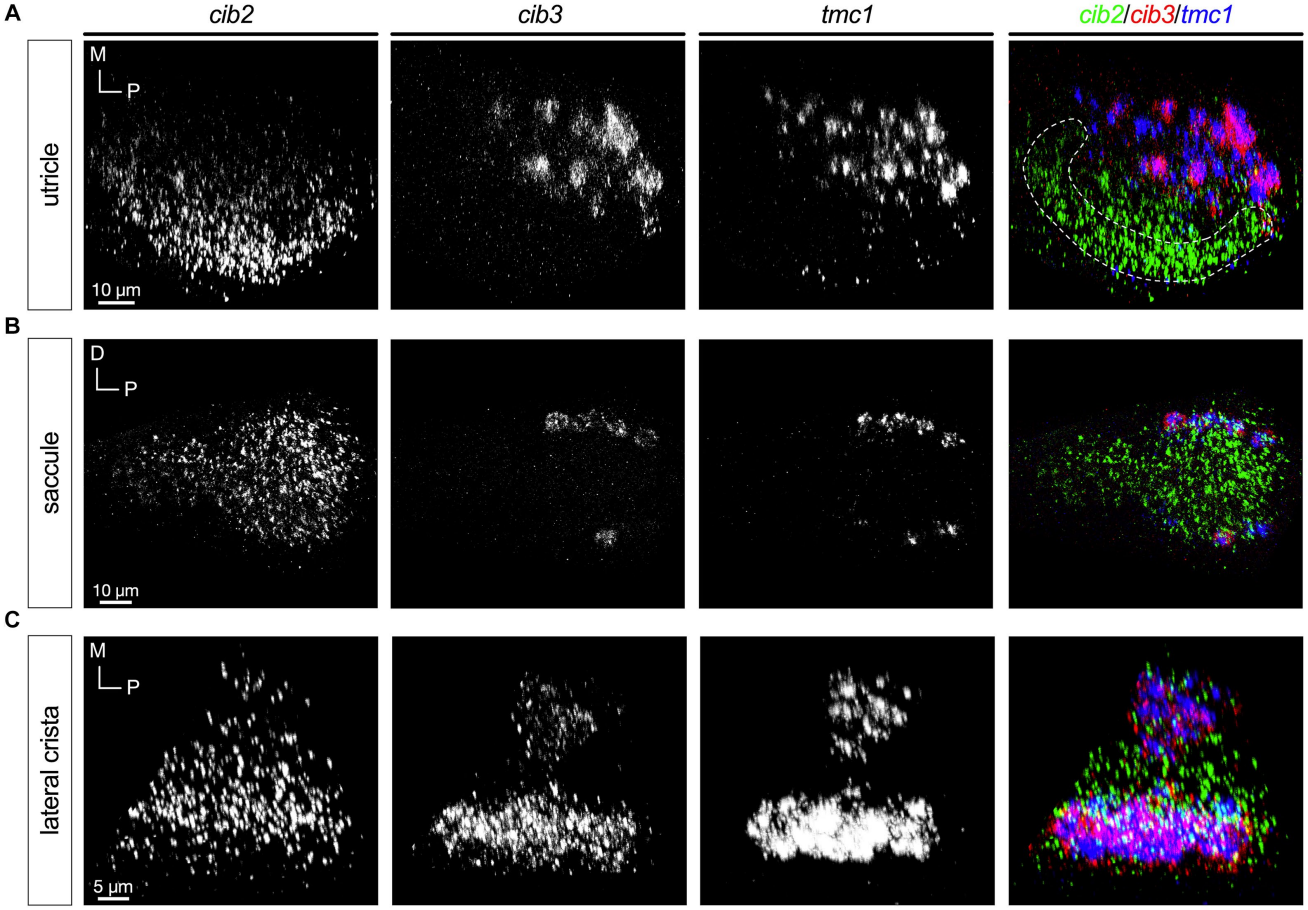
Expression of *cib3* correlates tightly with *tmc1* expression (5 dpf). In merged images, fluorescent label for *cib2* transcripts is shown in green, *cib3* in red, and *tmc1* in blue. **(A)** Single channels and merged image for utricular hair cells. **(B)** Single channels and merged image for saccular hair cells. **(C)** Single channels and merged image for ampullary hair cells of the lateral crista. The presumptive striolar zone of the utricle is outlined in **(A)**.

In all end organs we observed that the *cib2* and *cib3* genes had patterns that mirrored those of *tmc2a* and *tmc1*, respectively (Figure 7). *cib2* was broadly expressed like *tmc2a* whereas *cib3* was confined to *tmc1*+ cells (Figures 7A–C). These results suggest a preference for specific combinations of Tmc1/2 subunits and their closely associated Cib2/3 binding partners among different subtypes of hair cells.

**Figure 8:**
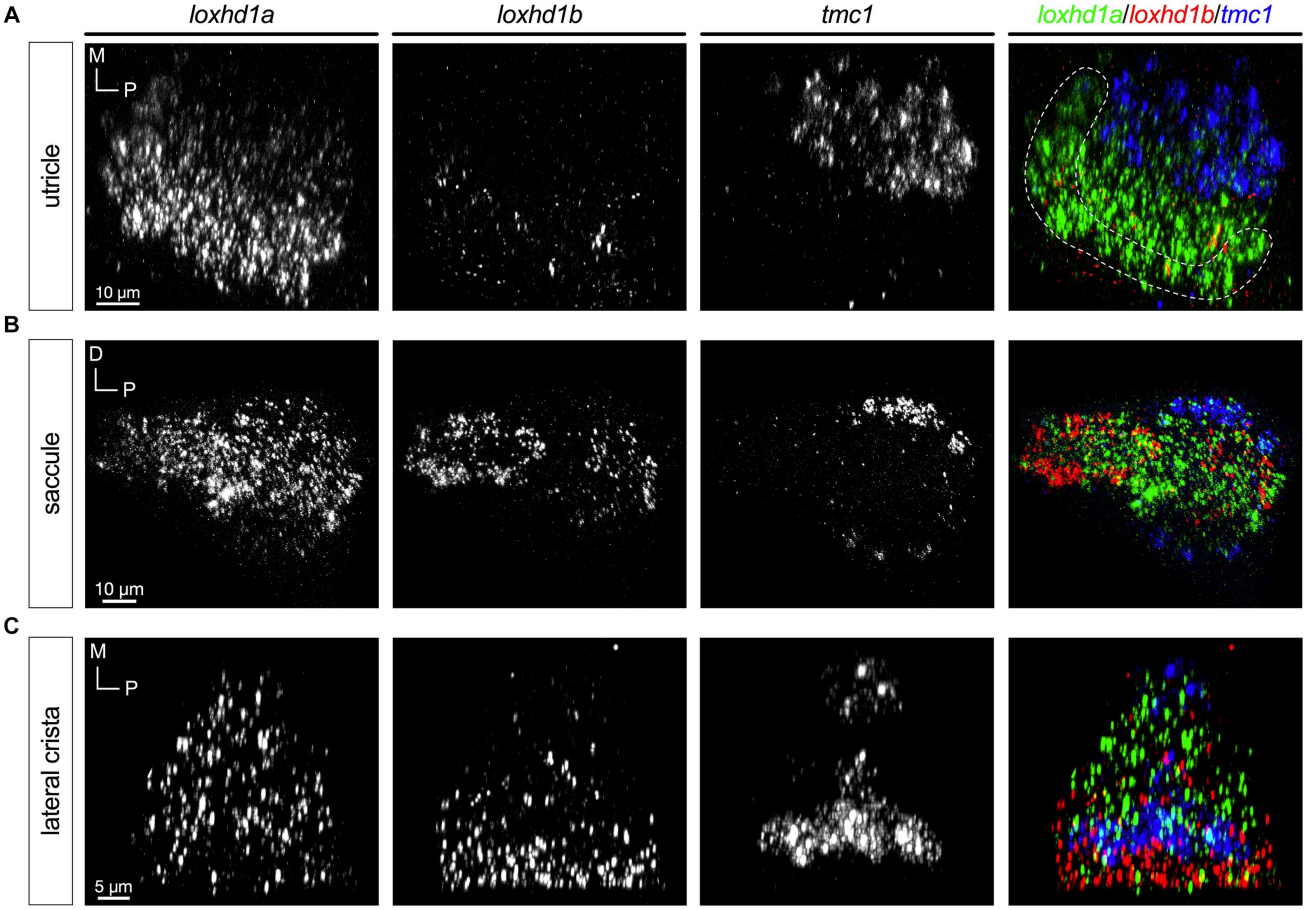
*loxhd1a* is enriched in striolar hair cells, whereas *loxhd1b* varies within each sensory organ (5 dpf). In merged images, fluorescent label for *loxhd1a* transcripts is shown in green, *loxhd1b* in red, and *tmc1* in blue. **(A)** Single channels and merged image for utricular hair cells. **(B)** Single channels and merged image for saccular hair cells. **(C)** Single channels and merged image for ampullary hair cells of the lateral crista. The presumptive striolar zone of the utricle is outlined in **(A)**.

The *loxhd1* paralogues *1a* and *1b* exhibited the most complex patterns (Figure 8). Like *tmc2a* and *cib2*, *loxhd1a* is broadly expressed in all hair cells with higher levels predominantly seen in the striolar regions (Figures 8A–C, left panels). In the utricle, *loxhd1b* is detectable at low levels in the striolar zone (Figure 8A). In contrast, *loxhd1b* is highly expressed in peripheral hair cells of the saccule’s anterior lobe, and in a small subset of hair cells of the posterior lobe (Figure 8B). We observed that hair cells that are positive for *tmc1* in the posterior lobe show much lower levels of *lohxd1a* and that *loxhd1b* is not detectable. In cristae, we observed expression of *loxhd1b* primarily in peripheral hair cells (Figure 8C).

**Figure 9:**
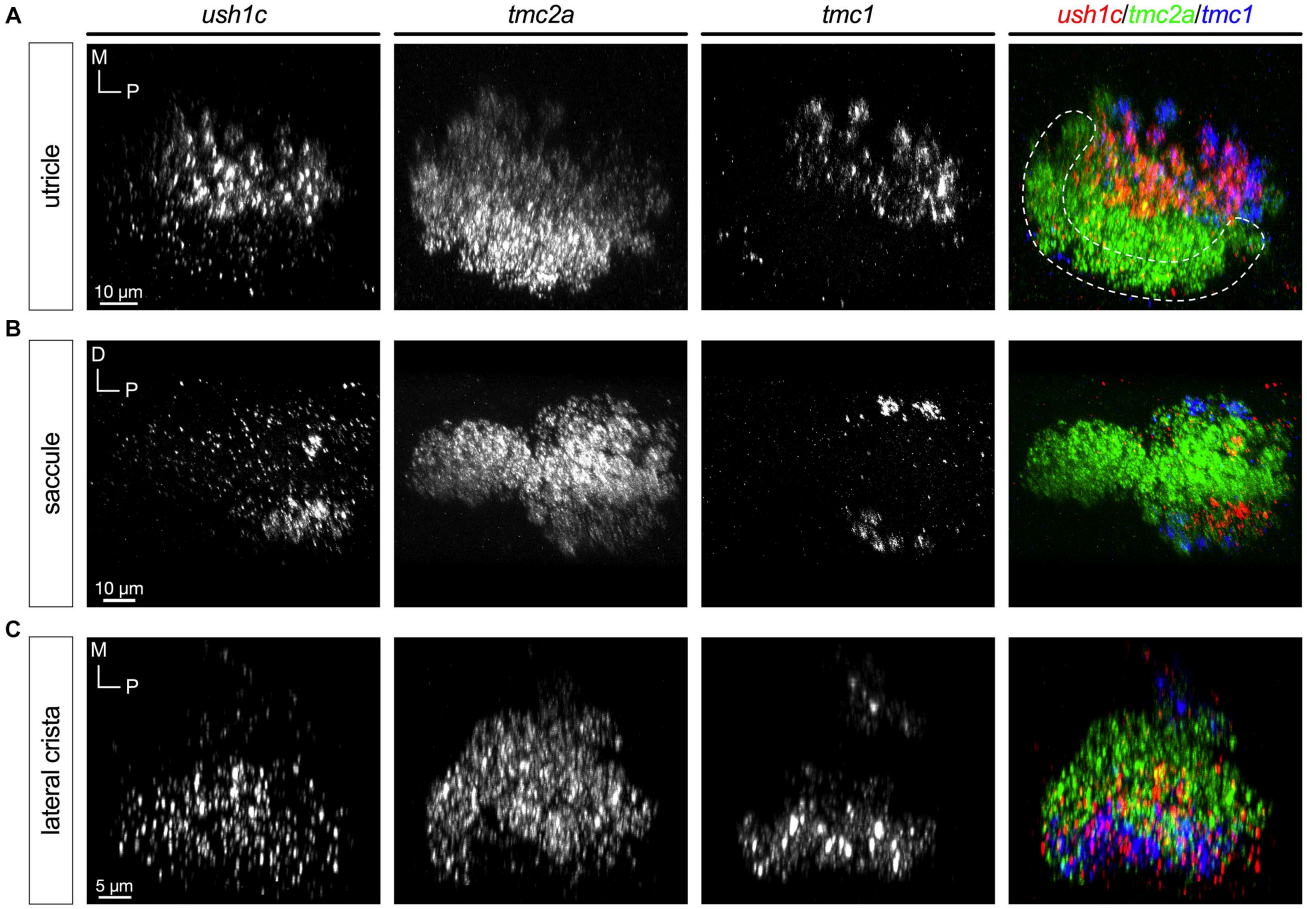
Expression of *ush1c* is enriched in peripheral or extrastriolar hair cells (5 dpf). In merged images, label for *ush1c* transcripts is shown in red, *tmc2a* in green, and *tmc1* in blue. **(A)** Single channels and merged image for utricular hair cells. **(B)** Single channels and merged image for saccular hair cells. **(C)** Single channels and merged image for ampullary hair cells of the lateral crista. The presumptive striolar zone of the utricle is outlined in **(A)**.

Finally, we examined the expression pattern of *ush1c* (Figure 9). Unlike the other mechanotransduction components, USH1C has been shown to localize to the upper tip link region in mouse hair cells (Grillet et al., 2009). Like *cib3* and *tmc1*, *ush1c* is expressed at high levels in extrastriolar or peripheral hair cells (Figures 9A–C); however, *ush1c* was also detected in central regions of each end organ, indicating that it is a universal component. Curiously, in the saccule *ush1c* signal was highest in a patch of ventral cells in the posterior lobe and in most of our specimens we noted a single central hair cell in the posterior lobe that expressed high levels of *ush1c* (Figure 9B).

In general, the subtype grouping identified via bioinformatic analysis was validated with RNA-FISH. One exception was the profiling of *tmc2b*, where scRNAseq data suggested low copy numbers of transcripts for this gene, whereas RNA-FISH suggested that expression was robust in specific cell types. These differences could be due to a high affinity of the *tmc2b* RNA probe sets used here or the relatively small number of hair cells captured in the RNAseq datasets that may be resolved with additional sequencing. Nevertheless, the RNA-FISH results validated the grouping of *tmc1/2* subtypes identified by bioinformatic analysis and demonstrated further differential expression of genes encoding essential components for mechanotransduction in hair cells.

## Discussion

Our data indicate that during initial stages of development (1 dpf), a brief period of mixed expression of the *tmc1/2* paralogues exists in nascent hair cells. Within 24 h, downregulation of one or two of the paralogues can occur, with several *tmc* subtypes emerging within the developing sensory organs. The most abundant subtypes include *tmc2a, tmc2a/2b*, and *tmc1/2a/2b*+ hair cells. These subtypes are further divided among the central or peripheral zones of each sensory organ. Triple expression of the *tmc1/2* paralogues is somewhat surprising given the dimer form of the *C. elegans* native TMC-1 complex (Jeong et al., 2022). Either multiple types of subunit complexes co-exist in hair cells or post-translational mechanisms may regulate the expression of the proteins. Post-translational regulation may provide an explanation for the lack of vital dye labeling of triple expressing gourd cells in the lateral cristae in *tmc1/2b* double mutants (Smith et al., 2020), however, further physiological and immunolabeling studies are needed to support this conclusion. Of the three *tmc1/2* paralogues, we found that *tmc2a* is the only gene that is expressed at high levels on its own and this cell type is abundant in central or striolar zones, whereas hair cells expressing high levels of *tmc1* are located strictly in peripheral or extrastriolar zones. In contrast to the other two *tmc1/2* family members, expression of *tmc2b* is variable among the sensory organs, but is largely restricted to peripheral cells like *tmc1*. One exception is the utricle, where approximately half of the striolar hair cells are positive for both *tmc2a* and *tmc2b*.

In mammals, the spatial distribution of *Tmc1/2* genes in vestibular hair cells has not been described in detail; however, in murine cochlear hair cells a switch in *Tmc* expression occurs during development with *Tmc1* replacing *Tmc2* expression. Consequently, *Tmc1* is required for mechanotransduction in cochlear hair cells at later postnatal stages, whereas *Tmc2* is not (Kawashima et al., 2011). These results are consistent with hearing loss in humans due to mutations in *TMC1* (Kurima et al., 2002; Makishima et al., 2004). Earlier studies in zebrafish indicated that unlike mice and humans, zebrafish do not rely on *tmc1* for hearing (Chen et al., 2020; Smith et al., 2020). However, there is indication that Tmc1 is sufficient for “inconsistent hearing” in zebrafish (Chen et al., 2020). In line with these studies, we found that very few hair cells express *tmc1* in the saccule, which is dedicated to hearing in fish. Why *tmc1* is confined to this very small population of extrastriolar hair cells, which persists at 15 dpf, and what role of *tmc1* plays in the saccule remains to be determined. Despite these differences in the preferred Tmc subunit for hearing among vertebrates, the overlapping expression of the *tmc1/2* paralogues during initial stages of development appears to be a common theme. How the final expression pattern is then established among the various subtypes of hair cells is an outstanding question.

A preference for one type of TMC subunit over another in a particular subtype of hair cells suggests that these channel subunits impart functional differences that are ideally suited for that subtype. In support of this idea, differences in unitary conductances and calcium permeability have been reported for single *Tmc* mutants in mice (Kim et al., 2013; Kim and Fettiplace, 2013; Pan et al., 2018; Fettiplace et al., 2022). Aside from selective zonal expression of the *tmc1/2a/2b* genes in the zebrafish inner ear, we also found further specialization of mechanotransduction components such as the *cib2* and *cib3* genes. CIB2 and CIB3 have been shown to physically interact with TMC1 and TMC2 and may offer some form of modulation of the mechanotransduction complex via the binding of calcium (Giese et al., 2017, 2023; Liang et al., 2021). In recent studies both *cib2* and *cib3* were shown to be required for mechanotransduction in cochlear hair cells and vestibular hair cell function in fish and mice (Liang et al., 2021; Giese et al., 2023; Wang et al., 2023). Furthermore, modeling of TMC1/2 and CIB2/3 complexes suggest that different combinations may give rise to different conductances (Giese et al., 2023).

In addition, we examined the expression of *ush1c* and *loxhd1a/1b* genes based on the subgroups that emerged in bioinformatic analyses. USH1C is a scaffold protein that has been shown to bind to the cytoplasmic tail of CDH23 and is thought to connect the CDH23 tip link to the actin cytoskeleton at the upper end of the tip link between stereocilia (Grillet et al., 2009; He et al., 2019). With respect to LOXHD1, mutations have been shown to abolish mechanotransduction in murine cochlear hair cells (Trouillet et al., 2021); however, a functional role in vestibular hair cells is not apparent (Schwander et al., 2007). In addition, a caveat to our RNA-FISH experiments with *ush1c* is that our probes are not selective for the b isoforms of *ush1c*, which is specifically required for mechanotransduction in mouse hair cells (Grillet et al., 2009). Our data, nevertheless, indicate that the *loxhd1a* and *ush1c* genes are broadly expressed in the zebrafish inner ear, in patterns that resemble those seen with *tmc2a* and *tmc1*, respectively. As with *tmc2a*, *loxhd1a* is enriched in striolar hair cells in the macular organs. In contrast, *ush1c* is expressed at much higher levels in extrastriolar hair cells, similar to *tmc1* in both the utricle and saccule. The high level of *ush1c* in macular extrastriolar hair cells is quite striking, if not unexpected. The pattern of *loxhd1b* is also highly specialized with almost negligible levels in the utricle and higher levels in peripheral hair cells of the saccule and lateral crista, albeit with the distinction of not being expressed in any extrastriolar hair cell that are positive for *tmc1* in the saccule. With respect to lateral cristae, zonal differences in *ush1c* and the *loxhd1* paralogues are less distinct. The expression patterns of the zebrafish homologues reported here are intriguing, and further investigation is required to determine how these differences in expression may lead to functional differences among hair cell subtypes.

In sum, our results reveal that a refinement in *tmc1/2* subunit expression occurs during early development in zebrafish inner ear hair cells. During maturation, subpopulations of hair cells either maintain expression of a combination of the *tmc1/2* paralogues or they predominantly express *tmc2a* alone. These subpopulations of hair cells are largely split between two zones of the neuroepithelium, with *tmc2a* primarily expressed in central or striolar zones and *tmc1/2b* expressed in the periphery or extrastriola of each sensory organ. Moreover, specific expression of the interacting “auxiliary” subunits of the mechanotransduction complex encoded by *cib2* and *cib3* is particularly evident in the case of *cib3*, which is selectively expressed in *tmc1*+ hair cells. These data suggest that distinct combinations of the mechanotransduction components are optimized to fulfill the specific functions of hair-cell subtypes.

## Data availability statement

The single-cell RNA sequencing dataset that was utilized in the study comes from the Danio-cell database (Sur et al., 2023). The data can be download via the NCBI Gene Expression Omnibus with accession number: GSE223922. The raw HCR data will be made available by the corresponding author upon request and a searchable database of the transcriptomic data is available at https://innerear.shinyapps.io/zebrafish_hair_cells.

## Ethics statement

The animal study was approved by Stanford University Laboratory Animal Care (APLAC) - Research Compliance Office. The study was conducted in accordance with the local legislation and institutional requirements.

## Author contributions

ES: Conceptualization, Writing – review & editing, Investigation, Methodology. PS: Conceptualization, Investigation, Methodology, Writing – review & editing, Data curation, Validation, Visualization. SY: Investigation, Methodology, Visualization, Writing – review & editing, Writing – original draft. DR: Writing – review & editing, Conceptualization, Funding acquisition, Supervision. TN: Conceptualization, Funding acquisition, Supervision, Writing – review & editing, Project administration, Visualization, Writing – original draft.
